# Prevalence and incidence of toxoplasmosis: a retrospective analysis of mother-child examinations, Styria, Austria, 1995 to 2012

**DOI:** 10.2807/1560-7917.ES.2016.21.33.30317

**Published:** 2016-08-18

**Authors:** Christian Berghold, Sereina Annik Herzog, Heidelinde Jakse, Andrea Berghold

**Affiliations:** 1 Labor Dr. Berghold, Graz, Austria; 2 Institute for Medical Informatics, Statistics and Documentation, Medical University of Graz, Graz, Austria; 3 Both authors contributed equally as first authors; 4 Mother-child booklet service, Styrian Health Insurance (Steiermärkische Gebietskrankenkasse), Graz, Austria

**Keywords:** toxoplasma, toxoplasmosis, antenatal screening, food-borne infections, pregnancy, protozoal infections

## Abstract

In Austria, mandatory screening for the prevention of congenital toxoplasmosis stipulates a serological test for antibodies against *Toxoplasma gondii* as early as possible in pregnancy. In the case of a seronegative result, subsequent tests at intervals of 8 weeks are requested. We analysed serological data from Styria, an Austrian federal state, to determine the seroprevalence and incidence of *Toxoplasma* infections. The study included 353,599 tests from 103,316 women during 158,571 pregnancies from 1995 to 2012. The age-adjusted seroprevalence decreased from 43.3% in 1995 to 31.5% in 2012, with a yearly decline of 0.84% (95% confidence interval (CI): 0. 79 -0.88). The intergravid incidence showed an annual decrease of 4.2%. The average yearly incidence of intragravid and intergravid seroconversions was 0.52% (95% CI 0.45–0.61) and 0.72% (95% CI 0.67–0.77), respectively. If the difference between these rates (p < 0.001) can be explained by the effect of primary prevention such as avoiding raw meat and taking hygiene precautions when encountering cats or preparing vegetables, only ca two of seven (28%) infections were avoided by hygiene measures taken by pregnant women. Primary prevention may therefore have its limits.

## Introduction

Primary maternal infection with *Toxoplasma gondii* during pregnancy can lead to prenatal infection of the unborn child, and vertical diaplacental transmission of *Toxoplasma* can seriously damage the embryo. Some prenatally infected children, asymptomatic at birth, can develop retinochorioiditis and other sequelae months or years later [1]. Since 1975, Austria has run prenatal screening for the early detection and treatment of toxoplasmosis, with the first test for *T. gondii* as early as possible in pregnancy [2]. If antibodies against these parasites are detected, the sample is further tested for specific IgM antibodies. A negative IgM report indicates a late latent infection that poses no threat for the current pregnancy. When a woman tests positive for IgM, the actual time of infection is determined as precisely as possible with special tests (avidity test, IgM immunosorbent agglutination assay, etc.). If there is still a suspicion of a primary infection in pregnancy, treatment according to the Austrian guideline is begun [2,3].

When the first test fails to show antibodies, the Austrian screening programme, which is part of the check-ups specified in the mother-child booklet (MCB), calls for further tests at 8-week intervals until the birth of the child. Development of specific antibodies to *T. gondii* in the further course of pregnancy is positive proof of a primary infection during pregnancy. Seroconversion is an indication for treatment. In recent years, a number of studies and meta-analyses have been undertaken to evaluate the effectiveness of antiparasitic treatment in pregnant women with *Toxoplasma* infections, but the results are inconclusive [4-6].

Evaluation of the screening programme for toxoplasmosis depends not only on the assessment of the effectiveness of treatment but also on a good understanding of the epidemiology of the disease. There are large variations in the seroprevalence and incidence of toxoplasmosis throughout the world. Countries and areas with low or very low incidence include the United States and northern European countries such as Norway, but also south-east Asia and the Sahel Zone [7]. In recent decades, there has been a clear decrease in the seroprevalence of latent infections, especially in industrialised countries [8]. A study in the United States of native-born inhabitants aged 12–49 years covering the years 2009–2010 produced an age-standardised seroprevalence of 6.7%, compared with 9% in 1999–2004 and 14.1% in 1988–1994 [9]. Factors that influence the probability of a human infection with *T. gondii* include climatic conditions in the region or country, nutritional habits of the inhabitants, the degree of development and the infection rates of the local cat population. Cats as definite hosts of *T. gondii* are able to shed oocysts through faeces. A moderate seroprevalence of 30–50% of persons with a latent infection is assumed in middle and southern Europe [7]. In Austria, a local study covering 2000–2007 showed a moderate seroprevalence of 31% in pregnant women [10]. In France, the average seroprevalence of latent infections among pregnant women was calculated as 54% in 1995 and decreased to 44% in 2003 [11]. Seroprevalence is highest in the moist tropical countries of South America and in tropical Africa.

There are few longitudinal cohort studies on the epidemiology of *Toxoplasma* infections. In an area with an implemented screening programme and centralised laboratory diagnostics, as is the case in two of the federal states in Austria, large-scale data analysis is possible. Styria, one of the nine federal states in Austria, has a population of 1.2 million. In Styria, *Toxoplasma* tests for pregnant women are usually processed in a central facility, the MCB service of the Styrian Health Insurance (Steiermärkische Gebietskrankenkasse or Stmk. GKK), where they record ca 9,000 pregnancies per year, representing 80–90% of all births in Styria.

The aim of this study is to determine the development of seroprevalence of latent *Toxoplasma* infections in pregnant women in Austria, a central European country, with direct calculation of the incidence of seroconversion during and between pregnancies in the period 1995–2012. It is assumed that differences between intragravid and intergravid seroconversion rates are due to the effects of primary prevention, such as avoiding raw meat and taking hygiene precautions when dealing with cats and vegetables. Since reliable data on adherence to the check-up schedule in the MCB are important for the evaluation of the screening programme, the number of seronegative women who had at least two follow-up tests will be determined to detect any changes that occurred over the years.

## Methods

Our retrospective cohort study was approved by the Institutional Review Board of the Medical University of Graz; Austria (EK Nr.: 26–031 ex 13/14).

The analysis was based on results of *Toxoplasma* tests done by the MCB service in the period from 1 January 1995 to 31 December 2012 that were exported from Stmk. GKK’s laboratory data system. Identifying fields that fell under data-privacy laws were pseudonymised. The dataset was rigorously tested for consistency and plausibility.

### Antibody screening

During the period covered by the study, three different test systems were used to screen for antibodies against *T. gondii*: from 1 January 1995 to 18 June 2006, the MCB service used slides coated with *T. gondii* for indirect immunofluorescence test (IIFT). From 19 June 2006 to 5 December 2010, the determinations were made with the automated Vidia Toxo IgG System from bioMérieux. Since 6 December 2010 *Toxoplasma* IgG has been determined automatically using the Architect System from Abbott Diagnostics. *Toxoplasma* IgM test to confirm seroconversion was determined with the following systems: from 1 January 1995 to 18 June 2006, the automated Vidas Toxo IgM System from bioMérieux; from 19 June 2006 to 5 December 2010, the automated Vidia IgM assay from bioMérieux; and since 6 December 2010, the automated Architect System IgM assay from Abbott Diagnostics.

### Defining pregnancies

The dataset included information on week or month of gestation when examination took place. Since there was a unique ID for each woman, examinations could be identified as pertaining to a single woman. For 80% (82,446/103,316) of women and their examinations, complete and coherent information on gestational age was available. Sometimes information on gestational age was not provided for each examination but from the time course (date of examinations) it was obvious that they pertained to one particular pregnancy (16% of women and their examinations). If there was no information for several examinations in sequence or contradictory information on gestational age, we classified them as belonging to the same pregnancy when they took place within a time window of 300 days. Women with no information at all about week or month of gestation for all their examinations were excluded from the dataset.

### Statistical analysis

The first examinations per pregnancy for a woman were used to determine the seroprevalence of latent toxoplasmosis aged between 15 and 44 years. Positive and borderline results were considered evidence of a past infection. A random-sample follow-up study at a Competence Centre for Toxoplasmosis (Vienna General Hospital) comparing sera with borderline test results and the gold standard, the Sabin-Feldmann dye test, showed that of 30 borderline sera, only one showed no antibodies. Although none of the older results could be rechecked, it seemed acceptable to also classify them as antibody positive [7]. The proportions of latent infections calculated in this way are shown both as yearly age-corrected seroprevalence and per age group and year. Additionally, a separate analysis was made for the time periods when the IIFT assay and ELISA assays were used.

The yearly incidence of new infections was calculated in three ways. For better comparability, the incidence, when not stated otherwise, always refers to seronegative women, i.e. the population of women at risk for infection, and not all women in the dataset.

(i) A frequently used procedure for estimating the incidence is based on the calculation of the increase in seroprevalence per year of life [7,12].

(ii) Seroconversions in pregnancies. These were women who did not have antibodies against *T. gondii* at the MCB check-up at the start of the pregnancy but at a later examination in the same pregnancy. Only those cases with a first negative examination after 1 January 1995 were considered. It is generally accepted that new *Toxoplasma* infections are associated with specific IgM antibodies [13], so that an intragravid seroconversion requires the presence of specific IgM antibodies. These intragravid seroconversions were related to the total risk period that was monitored on the basis of all MCB examinations at the MCB service within a pregnancy.

(iii) Infections that developed between pregnancies. Due to the extensive data available, it is possible to identify new infections (here as intergravid seroconversions as distinct from intragravid seroconversions, see above) between pregnancies. We analysed data from women who had two or more pregnancies and whose last examination in her first MCB-documented pregnancy failed to show antibodies to *Toxoplasma*. The woman’s entire dataset was examined for later *Toxoplasma* tests during any subsequent pregnancy. If the first test of a pregnancy showed antibodies and the last test from the previous pregnancy did not, then the infection must have occurred in the period between these two tests. The time point of these infections was defined using the following sampling procedure with 10,000 replications: for each woman with an intergravid seroconversion, we uniformly sampled the date of seroconversion from the time period between the two tests. The total risk time between all test pairs (neg-neg, neg-pos) formed the basis for the incidence calculation. The median number of intergravid seroconversions per year over all replications were used to calculate the incidence for 3-year intervals and for the total incidence over all 18 years.

For seroprevalence and incidence, 95% confidence intervals (95% CI) were calculated based on the exact method under a binomial distribution. Age adjustment was done using direct standardisation with the 1995 female census population of Styria as reference population and using 5-year age groups from 15 to 44 years. Changes in seroprevalence over time were analysed with a logistic regression model. To estimate the incidence based on the increase of seroprevalence per year of age, a binomial regression model with identity link was used with the implicit assumption that force of infection did not change over the years. Poisson regression models were applied to estimate changes in incidence (based on intragravid and intergravid seroconversion) over time. Poisson rates were compared with an exact test. A p-value < 0.05 was considered statistically significant. All analyses were performed using the R statistical software (version 3.2.2) [14].

## Results

**Figure 1:**
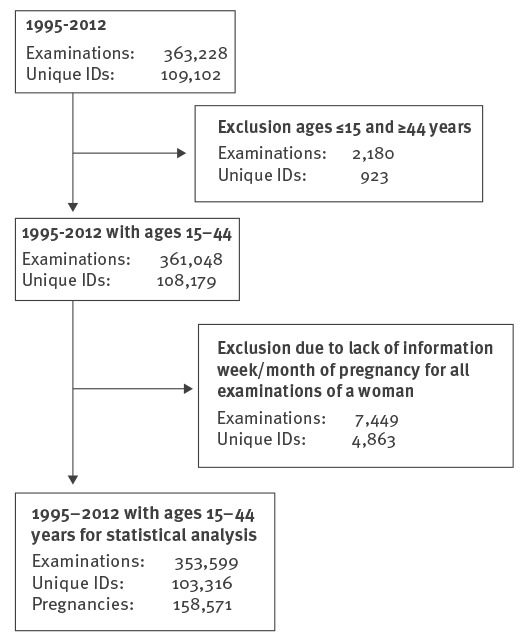
Flowchart explaining the selection of data for the analysis of prevalence and incidence of toxoplasmosis, mother-child examinations, Styria, Austria, 1995–2012

For the study period of 1995–2012, there were 363,228 screening tests. After application of the inclusion and exclusion criteria, 353,599 screening tests were analysed from 103,316 women and their 158,571 pregnancies (Figure 1). The median age in all pregnancies (first examination) was 27.2 years (interquartile range (IQR) 23.9–30.7) in 1995 and 29.8 years (IQR 25.9–33.5) in 2012.

Unique ID: each woman had a unique code assigned, so that examinations could be identified as pertaining to that particular woman.

### Seroprevalence of *Toxoplasma* infections in pregnant women

**Figure 2:**
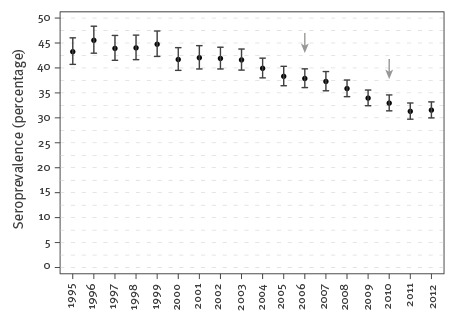
Annual age-adjusted seroprevalence of *Toxoplasma gondii* infections in pregnant women aged 15–44 years, Styria, Austria, 1995–2012 (n=158,571)

The annual age-adjusted seroprevalence is shown in Figure 2.

Age adjustment was done using direct standardisation with the 1995 female census population of Styria as the reference population. Arrows indicate the change from IIFT to Vidia (2006) and from Vidia to Architect (2010).

**Figure 3:**
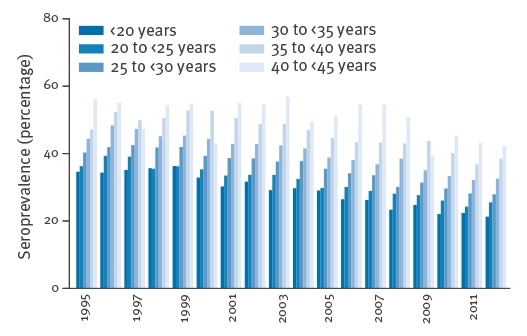
Yearly seroprevalences of pregnant women aged 15–44 years with antibodies to *Toxoplasma gondii*, by age groups, Styria, Austria, 1995–2012 (n=158,571)

In 1995, 43.3% (95% CI: 40.7–46.0) of the pregnant women showed antibodies against *T. gondii* at the first examination per pregnancy; in 2012 the seroprevalence was 31.5% (95% CI: 30.0–33.2). In the 18 years studied, the seroprevalence of women with latent infections on average decreased by 0.84% yearly (95% CI: 0.79–0.88, p < 0.001). Furthermore, we made a separate analysis of the time periods for the IIFT assay and ELISA assays (Vidia and Architect). In the time period 1995–2006, there was a decline in seroprevalence of 0.56% (95% CI: 0.46–0.66, p < 0.001) yearly, while from 2006 to 2012, there was a 1.20% annual decline (95% CI: 1.00–1.40, p < 0.001). Figure 3 shows the yearly seroprevalence according to age groups of pregnant women who showed antibodies to *T. gondii* at their first examination in pregnancy. Seroprevalence is shown to increase with age (p < 0.001, linear test for trend).

The first test per pregnancy is considered.

### Incidence of new infections

Calculation of the age-dependent increase in seroprevalence during the 18-year study period showed an average estimated incidence of 0.85% new infections per year (95% CI: 0.81–0.89).

**Figure 4:**
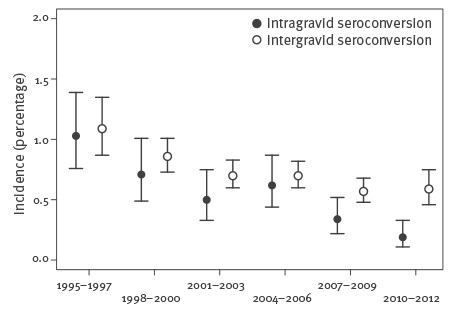
*Toxoplasma gondii *infections:**incidence of intra- and intergravid seroconversions in women aged 15–44 years at three-year intervals, Styria, Austria, 1995–2012

From 1995 to 2012, 167 intragravid seroconversions were registered. There was complete agreement between the cases extracted from the database and the MCB service’s internal documentation. The observation period was 31,940 person-years, for an average yearly incidence of 0.52% (95% CI 0.45–0.61) for seronegative women, see Table 1 and Figure 4.

**Table 1:** *Toxoplasma gondii* infections: incidence of intra- and intergravid seroconversions for in women aged 15–44 years at three-year intervals, Styria, Austria, 1995–2012

Year	Intragravid seroconversion		Intergravid seroconversion	
	**Incidence (%)**	**95% CI**	**Incidence (%)**	**95% CI**
1995–1997	1.03	0.76–1.39	1.09	0.87–1.35
1998–2000	0.71	0.49–1.01	0.86	0.73–1.01
2001–2003	0.50	0.33–0.75	0.70	0.60–0.83
2004–2006	0.62	0.44–0.87	0.70	0.60–0.82
2007–2009	0.34	0.22–0.52	0.57	0.48–0.68
2010–2012	0.19	0.11–0.33	0.59	0.46–0.75
Total	0.52	0.45–0.61	0.72	0.67–0.77

CI: confidence interval.

The incidence (%) of intra- and intergravid seroconversions with the corresponding 95% confidence intervals.

The general infection risk for a pregnant woman can be determined if the probability of infection is assumed to be the same throughout pregnancy. With an average seroprevalence for the eighteen years of latent infections of 39.0% (95% CI: 38.5–39.4) and duration of pregnancy of 0.75 years, a woman’s risk, regardless of toxoplasmosis status, of acquiring a new infection with *T. gondii* is 0.24% per pregnancy (range: 0.20–0.28). This means that a new infection with *T. gondii* can be expected in one in 409 pregnancies.

Among 42,816 women studied for possible changes in *Toxoplasm*a status between pregnancies, i.e. having two or more pregnancies, there were 732 cases of intergravid seroconversion (Table 1). The average annual total incidence was 0.72% (95% CI: 0.67–0.77) in seronegative women. The Poisson regression gave an average yearly reduction of 4.2% (95% CI: 2.5–5.8) of intergravid seroconversions during the period 1995–2012.

Comparison of the two incidences gave a significant difference between intergravid seroconversions (0.72%) and intragravid seroconversions (0.52%) (p < 0.001; comparison of Poisson rates).

### Primarily seronegative pregnant women who presented for at least three examinations

Table 2 comprises the percentage of women attending the MCB service at Stmk. GKK who underwent at least two follow-up tests due to an initial negative test (i.e. at least three tests during a pregnancy). The classification by year is based on the date of the first examination during a pregnancy).

**Table 2:** *Toxoplasma gondii *infections: primarily seronegative pregnant women who presented for at least three examinations, Styria, Austria, 1996-2011

Year	Number	% of total
1996	2,235	31.0
1997	2,159	32.2
1998	2,114	32.6
1999	2,107	33.9
2000	2,229	35.5
2001	2,379	37.3
2002	2,525	41.2
2003	2,507	39.9
2004	2,649	41.5
2005	2,870	43.5
2006	3,220	54.4
2007	3,469	62.4
2008	4,046	60.0
2009	4,177	62.7
2010	4,332	64.8
2011	4,515	67.8

## Discussion

Conforming to a trend in other industrialised countries, the proportion of women in the state of Styria, Austria, who already have a latent infection with *T. gondii* before pregnancy has been decreasing. In 1995, 43.3% (95% CI: 40.7–46.0) of pregnant women showed antibodies to *T. gondii* at the first examination per pregnancy; in 2012 the figure was 31.5% (95% CI: 30.0–33.2). In the 18-year study period, the seroprevalence of latent infections in pregnant women decreased by 0.84% (95% CI: 0.79–0.88) annually. When screening began in 1975, about half of pregnant women showed antibodies against *T. gondii* [15]. When the total seroprevalence of latent infections does not change over the years, the incidence of new infections can be estimated from the age-dependent increase in seroprevalence. Under the false assumption that seroprevalence stayed constant, an average new infection rate of 0.85% per year was calculated for the 18-year study period. In a paper published in 1994, Larsen and Lebech drew attention to the fact that with decreasing seropositive rates, the incidence calculation based on the age-dependent increase led to overestimation of the annual *Toxoplasma* infection rate [16]. If hypothetical human infections with *T. gondii* were to completely stop at a certain point in time, the age-dependent increase in seroprevalence would persist for many years. In this hypothetical model the persisting increase would lead us mistakenly to suppose a still existing infection-rate.

The average yearly incidence of *Toxoplasma* infections in the 18-year study period in pregnancies (intragravid seroconversions) and between two pregnancies (intergravid seroconversions) was 0.52% (95% CI: 0.45–0.61) and 0.72% (95% CI: 0.67–0.77) respectively per year for seronegative women. There was an average decrease in annual intergravid incidence of 4.2% over the years of the study. There was a similar decrease in both the incidence during pregnancy and between pregnancies. At the end of the period of 2010–2012, the intergravid incidence was 0.59% (95% CI: 0.46–0.75). Therefore it is to be expected that the seroprevalence of latent *Toxoplasma* infections will continue to show a clear decrease. That means, however, that the number of women of childbearing age who are at risk of infection will increase. The net effect is that although primary infections in pregnancy are decreasing, prenatal toxoplasmosis will continue to pose a substantial threat to pregnant women and their children.

The main source of human *Toxoplasma* infections is seen to be consumption of undercooked meat and meat products [17]. Modern technology in meat processing may have reduced the degree of contamination, and hygiene measures such as strict rodent control and cats in livestock barns help to reduce infection pressure. Deep-freezing meat for a number of days also helps to kill *T. gondii* [7]. For some years, however, there has been a trend towards organic and free-range farming, and in such operations, there is a higher seroprevalence of infection [18].

House cats are another important link in the infection chain. A shift towards sterilised pet food products from supermarkets and the increasing tendency to keep these pets indoors may mean that there are fewer cats excreting oocysts.

The difference in the total incidences during and between pregnancies can be traced to the effect of primary prevention. Primary prevention includes no consumption of raw meat, avoiding contact with cats and strict hygiene when coming into contact with vegetables and garden soil. In many health systems, such as those in England and Wales and the United States [7,19], primary prevention is the only measure in place to prevent congenital toxoplasmosis. For methodological reasons, there is at present no clear evidence of general effectiveness and relevance of primary prevention [20].

The degree of effectiveness of primary prevention reflected by our study is disappointing. It mainly results from the different incidences (intragravid to intergravid) in the last 6 years of the study. The data indicate that only ca two out of seven infections were avoided by primary prevention. There is no general systematic primary prevention approach implemented in Austria. Gynaecologists usually give advice about risk factors of *Toxoplasma* infection at the time of blood sampling for *Toxoplasma* tests. A desirable side effect of the extensive screening plan with repeated blood sampling should have been to make women of childbearing age aware of the dangers of primary infection. Although raw or undercooked meat (pork, beef, mutton, chicken, etc.) is seen as the main source of *Toxoplasma* infection, there are many other possible sources. Drinking water or ingestion of oocysts while swimming in natural waters could also lead to infection [21]. More recently, undercooked shellfish such as mussels and oysters have been considered infection sources [7,22]. Considering this multiplicity of possible sources of infection, primary prevention may have its limits [1].

Adherence to the MCB guidelines with respect to follow-up examinations for *Toxoplasma*-negative women has improved noticeably over the years. In 2011, some 67.8% of seronegative women had at least three tests in the MCB service. In another state (Upper Austria) in 2007, 35.5% of seronegative pregnant women had three or more serological toxoplasmosis tests [10]. In a Viennese study that ran from May 2001 to December 2002, the authors reported that after giving birth, only 22.5% of women at risk for infection showed evidence of three or more tests for toxoplasmosis in their MCBs [23].

### Limitations

The problems with retrospective studies are well known. For this study, an extensive data collection covering a very long time period was available. Since different assays have different sensitivities and specificities [24], comparison of results over a long time period is complex. Therefore we made a separate analysis of the time periods when the IIFT and ELISA assays (Vidia and Architect) were used (Figure 2). There was a decline in seroprevalence in each of these periods. The decrease in seroprevalence was also confirmed by the decreasing incidences of intragravid and intergravid seroconversions. This in turn indicates that our results are highly plausible.

When dealing with serological results, there are always constellations that are difficult to interpret. That was also true with the assignment of intergravid seroconversions. 662 (90%) of the 732 ultimately defined intergravid seroconversions were unequivocal, but 70 uncertain cases were considered as intergravid seroconversion on the basis of all available information. The authors believe that the number of intergravid seroconversions represents the upper limit.

Due to missing data the correct assignment of examinations to a pregnancy was in rare cases uncertain. Repeating the analysis using only cases with secure classification, the results remained stable (data not shown). Despite these limitations, we have, for the first time, been able to directly measure the seroconversions and incidences of Toxoplasma infections and their dynamics over time.
